# Elementary School Teachers’ and Counselors’ Decisions on Referring Students for Evaluation: The Impact of ADHD Traits, Achievement, and Giftedness

**DOI:** 10.1007/s10578-024-01777-0

**Published:** 2024-11-28

**Authors:** Avital Tamsut, Hattem Asadi, Gal Nahum Sinai, Noa Saka, Yehuda Pollak

**Affiliations:** The Seymour Fox School of Education, Hebrew University of Jerusalem, Jerusalem, Israel

**Keywords:** ADHD, Achievement, Giftedness, Teacher referral, Vignettes

## Abstract

The present study examined the effect of ADHD-related traits, academic-achievement level, and giftedness label on elementary school teachers’ and counselors’ referral recommendations for assessment. 532 teachers and counselors were presented with one of 12 vignettes describing a hypothetical pupil. Participants were asked to report the likelihood they would refer the pupil for ADHD diagnosis and address them during a high-level interdisciplinary school-team meeting (HISTM). High ADHD-related traits (effect size 0.359) and low academic-achievement (effect size 0.070) and their interaction were significantly related to a higher likelihood of referral. Further analysis revealed that lower academic achievement was related to a higher likelihood of referral only when ADHD-related traits were not indicated (p < .005). The status of giftedness label was not found to be significant (p > .05). These findings indicate that mainly ADHD-related traits and, to a lesser degree, low academic-achievement influence teachers’ decisions to refer pupils for ADHD diagnosis and address them in HISTM.

## Attention Deficit Hyperactivity Disorder

Attention Deficit Hyperactivity Disorder (ADHD) is a neurodevelopmental disorder characterized by a persistent pattern of symptoms of inattention and or hyperactivity-impulsivity. Symptoms must appear before age 12 but may continue to diminish a person’s academic, social, and occupational performance throughout their life span (Diagnostic and Statistical Manual of Mental Disorders- DSM-5).

## ADHD-Related Traits: Behavioral, Social & Academic Characteristics

ADHD affects and may impair multiple aspects of day-to-day life. Children with ADHD are in increased danger of having social, scholastic, and vocational difficulties [29, 44].

In the classroom, pupils with ADHD exhibit lower school engagement, reflected by more off-task behavior, difficulty waiting for their turn, disobedience, and poorer student–teacher relationship quality [6], Johnson at al. [18, 25, 43, 58, 61]. Extensive research has found ADHD to be associated with poorer academic outcomes, such as lower grades on report cards, lower GPA & GCSE scores, class retainment, and high school dropout [12, 13, 22, 26, 29].

## Giftedness

Over time, the latent concept of giftedness has evolved. Early concepts emphasized a high general ability (score 130 or higher on an intelligence test) as the core definition of giftedness. Influenced by broader theories of intelligence, the next phase of research emphasized domain-specific excellence as the core of giftedness. In the third stage, Renzulli’s three-ring theory significantly impacted research [39]. Giftedness was defined through a system framework in which various psychological processes co-exist and must work together. Ecological-environmental processes have influenced more recent definitions and view giftedness as a more dynamic and ever-developing talent [1, 7, 15, 32], Stoeger et al. [50]).

The concept’s evolution is evident in the definitions of giftedness adopted by institutes and official government policies, including *The National Association for Gifted Children (NAGC),* the *US Ministry of Education through the Jacob K. Javits Gifted and Talented Students Education Act,* the UK* Ministry of Education, and Israel’s Ministry of Education.* All have defined giftedness by incorporating general ability, specific ability, and creativity while addressing gifted children’s unique educational, social, and emotional needs.

## ADHD and Giftedness

Gifted children and children with ADHD exhibit some similar behavioral characteristics. It has been argued that this overlap leads to a misdiagnosis of some gifted children as coping with ADHD [10, 17, 40]. One of the frameworks addressed in the literature examining similarities of characteristics associated with giftedness and ADHD is *Overexcitabilities* – a term stemmed from Dabrowski’s personality theory of positive disintegration [10, 17, 24, 40]. Overexcitabilities can be categorized into five dimensions representing personal characteristics associated with how a person processes a vast number of environmental stimuli: *psychomotor, sensual, intellectual, imaginational, and emotional* [41].

Rinn & Reynolds [41] describe the manifestation of overexcitabilities in day-to-day life. They construe the presentation of imaginational, psychomotor, and intellectual overexcitabilities:* "Imaginational overexcitability* is expressed through vivid and detailed daydreams or fantasies, inventiveness, use of imagery, and verbal utilization of metaphors. *Psychomotor overexcitabilities* are expressed by extreme activity or displays of frenetic energy. An individual with psychomotor overexcitability might have rapid speech, display impulsiveness, and appear to be in constant motion in some manner, such as wiggling his or her feet or hands. *Intellectual overexcitabilities* reflect a deep need for knowledge and problem-solving. Intellectual overexcitabilities may be expressed by persistently asking questions, critical observation, and use of theoretical analysis" [41], p. 40).

It can easily be seen why some behaviors gifted pupils exhibit might be wrongly attributed to ADHD. For instance, a gifted child might seem inattentive as they imagine the solution to a complex problem or try to master a task that takes others longer. They might make careless mistakes or not follow instructions as they are bored doing a repetitive task or have quickly mastered the skill. Another reason might be that gifted children find it challenging to persist on tasks they deem irrelevant or beneath their abilities. Excessive movement and verbalization may occur when gifted children pursue their interests and are excited to explain their innovative ideas to others. Excessive movement can also occur when a gifted child is bored or is in an environment that does not challenge them enough [10, 17, 24, 27, 40].

Though similarities exist between behavioral characteristics associated with giftedness and symptoms related to ADHD, the causes for such behaviors and their breadth are different. For example, a gifted child might exhibit hyperactivity, but that hyperactivity will be focused and goal-orientated. In contrast, a child with ADHD will exhibit hyperactivity in many environments and will not be focused or goal-orientated [10, 24].

## Twice-Exceptional Children

The term *‘twice-exceptional*’ refers to individuals who, in addition to being identified as gifted, are diagnosed with a disability or impairment such as specific learning disabilities, ADHD, Autism Spectrum Disorder, etc. [59]. This subgroup shares the characteristics affiliated with giftedness and those affiliated with their specific disability.

In the case of ADHD, gifted pupils with ADHD have been found to show similarities to gifted pupils without ADHD regarding their abilities and creativity [24]. However, on tasks sensitive to executive functions impairments and distractibility, they do not perform as well as their gifted peers who don’t have ADHD [3, 24].

Gifted pupils who show a clinical level of ADHD symptoms have similar impairments in their school functioning to children with ADHD who are not gifted (i.e., with average intelligence). Exhibiting underachievement, problems initiating tasks, staying on task, and avoiding homework [5], Mulet and Rinn [34]).

Even though twice-exceptional children tend to have some impairments, the literature recognizes a twofold phenomenon known as the ‘*Masking Effect’*. The twice-exceptional individual’s disability may mask their giftedness, while their high abilities compensate for the impairments caused by their disabilities, resulting in under-identification of both their giftedness and disabilities [5, 24].

## The Important Role of Teachers in Diagnosing Pupils with ADHD

Although teachers are not qualified to diagnose ADHD, they have a vital role in the diagnosis process. A multi-method, multiple sources of information examining occurrences of the behaviors in multiple settings is recommended as the gold standard for diagnosing ADHD (DSM-V). In the case of diagnosing school-aged children, teachers are an essential source of information about a child’s behavior and functional impairment caused by ADHD. Teacher rating scales are one of the main tools clinicians use to determine if a diagnosis of ADHD or other disorders is warranted (Nelson et al. [36]).

Teachers tend to consider more students as showing clinical levels of ADHD symptoms than the actual prevalence of the disorder [9, 21]. Teachers’ ratings on ADHD behavioral scales were influenced by a pupil’s gender, age, parental education, relationships with the teacher, and experience. Teachers were also influenced by the quality of the pupil’s peer relations and their relationships with the child [9, 21, 51].

In the case of gifted pupils, research is inconclusive concerning what effect the label of giftedness has on teachers’ ratings for ADHD symptoms. Some research found that teachers’ ratings of ADHD symptoms among gifted pupils were higher than for non-gifted pupils. In contrast, others have found teachers to rate gifted children with lower levels of hyperactivity and attention problems than their parents [5, 24, 46, 55].

Several studies found a correlation between referral for evaluation and initiating interventions with teachers’ knowledge of ADHD symptoms, causes, and treatment. Teachers were also highly influenced by the knowledge they gained from the media, family, and friends. Their attributions regarding referral were influenced by social norms and experiences with pupils with ADHD and their personal beliefs that referral would lead to an intervention, either by the referred child’s parents or the school [23], Rinn and Nelson [40].

While academic achievement was found to influence teachers’ referral decisions, it did not have as much influence as other factors, such as the pupil’s peer relations [21]. Interestingly, teachers’ referral decisions have been found to be affected by the child’s general ability. As mentioned above, teachers have been found to rate gifted children as showing fewer hyperactivity and attention problems than children with lower IQ [46, 55]. Rommelse et al. [46] interpreted these findings to indicate that teachers attributed less severity to ADHD symptoms exhibited by gifted children. This body of research might explain Mullet & Rinn’s [34] findings that gifted pupils with ADHD tend to be identified later.

Research by McCoach, Siegle & Rubenstein [30] challenges the findings of Mullet & Rinn [34]. This research found that among a group of underachieving gifted students, based on teacher’s ratings, only 20% met the *DSM’s* criteria for ADHD diagnosis. Yet, teachers evaluated 50% of them as needing a formal clinical assessment for ADHD. Yet, they considered only 21.5% of students in the non-gifted group to require such an assessment.

Use of vignettes in research:

Vignettes have been widely used in the literature to assess factors influencing teachers’ attitudes, biases, inferences, and judgments in both the fields of ADHD and giftedness research [4, 11], Harttnet et al. [17, 28, 33, 37, 40, 48].

In their paper on using vignettes, Skilling, & Stylianides, [49] summarize the benefits and pitfalls of using vignettes in educational research. Vignettes have many benefits. If constructed carefully, they enable addressing issues that might not be easy or comfortable for the participants to address, whether due to emotional difficulties social or un politicly correct attributions.

One major pitfall of using vignettes to assess teachers’ practices is that their reaction or action chosen to the questions following the vignette presented may differ from the action they would take in real life. When researching teachers’ beliefs and understandings about a phenomenon, it is appropriate to use methodologies and methods that are situated in the cultural and social settings within classrooms [49]. Hence, vignettes must describe the phenomena targeted for the research in the relevant setting.

Research examining teachers’ attitudes towards children with ADHD using vignettes in which a label of ADHD was stated found mixed patterns of bias and stressors relevant to managing a child with ADHD in their class. The ADHD label led teachers to view the pupil as less intelligent and less favorable in terms of personality and behavior. Teachers perceived behaviors of friendships and classroom conduct as more disruptive when the child had a label of ADHD. They also reported experiencing more stress in managing these pupils compared to pupils exhibiting the same behaviors but without the ADHD diagnosis. However, teachers were more inclined to intervene on behalf of a diagnosed child [37].

As for gifted and twice-exceptional pupils, previous research mostly asked teachers to classify which diagnosis better fits a pupil presented in a vignette describing behaviors that could be attributed to ADHD, giftedness, or both. When teachers were forced to choose from a list of diagnoses that included giftedness as one of the options, they indicated giftedness as an option than when no list was provided [11], Harttnet et al. [17, 40].

## The Presents Study

Teachers hold a central role in their pupils’ educational, social-emotional development, and overall well-being. By bringing attention to symptoms and impairments, teachers have a crucial role in identifying children in their class who might need professional evaluation and specific interventions due to impairments caused by ADHD (Furzer et al. [14, 21], Lee and Olenchak [24, 40, 42, 47], Thielking and Terjesen [52]).

Most referrals of children for ADHD diagnosis occur during elementary school. In many cases, the child’s primary teacher is the first to notice the impairments in the child’s performance or behaviors and the need for a professional diagnosis and communicate them to the parents.

As discussed above, previous research has found conflicting findings as to the influence of factors such as ratings for the severity of ADHD symptoms, academic achievement, and giftedness on teachers’ decisions of referral for ADHD diagnosis [5, 24, 30, 46, 55]. Therefore, the effects of such variables on teachers’ referral decisions remain unclear. Considering teachers’ crucial role in these pupils’ lives, it is important to examine further the influence of these factors on teacher’s referral decisions.

Previous research has found vignettes to be an acceptable and reliable method used in educational research [49]. To our best knowledge, past research either gave a label of ADHD or asked teachers to decide what diagnosis, if any, best fits the described pupil. In the present study, we used vignettes to describe behaviors that could be attributed to both giftedness and ADHD. To better understand the effects of ADHD, academic achievement, and giftedness on teachers’ decisions to refer them for ADHD diagnosis, we specifically stated a label of giftedness in relevant vignettes.

The present study’s objective is to use an experimental design to examine the effects of three factors: ADHD-related traits (i.e., symptoms, school engagement, nonacademic impairments such as organization, peer relations, injuries, and tardiness), academic achievement level, and giftedness label, on teachers’ recommendations for referring pupils for a formal diagnosis of ADHD and for addressing them during a High-level interdisciplinary school team meeting (HISTM).

The present study aims to answer two main research questions:

1. To what extent would these three factors (a) ADHD-related traits, (b) academic achievement levels, and (c) label of giftedness affect teachers’ intent to refer pupils to assessment for ADHD and address them during a HISTM.
2. Would teachers’ decision to refer pupils to an evaluation for the diagnosis of ADHD differ from their decision to address them during a HISTM?

The manipulation of the three independent variables was translated into several research hypotheses: Both ADHD-related traits and academic achievement level will affect teachers’ decisions. Teachers will be more likely to refer a pupil for ADHD assessment and to address them during an HISTM when ADHD-related traits are indicated. Teachers will show more intent to refer and address pupils during an HISTM when a pupil’s academic achievement level is low compared to his peers.

In addition, giftedness will affect teachers’ perceptions and the referral of pupils differently depending on the pupils’ academic achievement. High achievement will be correlated with lower referral rates, while low achievement levels will be correlated with higher achievement rates in pupils labeled as gifted.

A secondary aim of this study was to examine if a giftedness label would affect teachers’ decisions given an identical description of the pupil’s performance.

## Methods

### Inclusion and Exclusion criteria

Inclusion criteria were established as completing the first two questions, which addressed the main research questions: (1) What is the likelihood that you would refer the pupil described to be evaluated for an ADHD diagnosis? (2) What is the likelihood that you would address the pupil described in an HISTM?

### Sampling Procedure

Participants were recruited using an individual self-selection sampling procedure. Participants were contacted in 2020 (during the outbreak of the COVID-19 pandemic) via social media: Facebook posts were posted in relevant groups, and direct personal messages (DM) were sent to individual members of WhatsApp groups dedicated to elementary school teachers and counselors. Each message or post included a short description and a link to the online research questionnaire.

General message posted on relevant Facebook groups:

"Elementary school homeroom teachers and educational counselors, I would greatly appreciate your help by answering a short research questionnaire (in the link provided). The research is being conducted as part of a thesis within the department of learning disabilities at the Hebrew University of Jerusalem. The questionnaire is anonymous and received the ethical board approval and the approval of the Ministry of Education."

DM WhatsApp message.

"Hello, my name is Avital Tamsut. We are both members of the WhatsApp group (name of specific primary teachers and school consolers group). I was wondering if you would be willing to help me (as a colleague) by answering a short research questionnaire intended for primary school homeroom teachers and school counselors.

Link to the research questionnaire.

The research is being conducted as part of a thesis within the department of learning disabilities at the Hebrew University of Jerusalem. The questionnaire is anonymous and received the ethical board approval and the approval of the Ministry of Education. "

Prior to the start of their participation, informed consent was obtained from each participant. Participants were not offered any personal or other gains for participating in the study.

## Participants

Five hundred and fifty-eight Israeli elementary school home-room teachers, teaching 1st through 6th grades, and primary (elementary) school counselors participated in the study. Of the sample, 26 participants did not complete the first two questions and were excluded from the study.

The final sample consisted of 532. The sample size was predetermined based on power calculation (G*Power 3.1.9.2), indicating that a sample of 500 is sufficient for identifying small-size main effects and interactions (f = 0.10) with a power of 0.9 and a significance level of 0.05. No stopping rules were employed.

Detailed demographic characteristics are presented in Appendix C. Chi-Square and ANOVA tests revealed no significant differences among participants in the distribution of gender and age across the 12 types of vignette conditions.

## Materials

### Vignette Development and Psychometrics

In this study, vignettes were developed in Hebrew based on DSM-5 criteria for ADHD, characteristics of gifted and twice-exceptional students reviewed in the literature [16, 33, 45]. Each vignette described a boy with peer relations difficulties (e.g., he is not one of the popular kids, and during recess, he often walks around alone and gets into conflicts with his classmates and some of his teachers). However, they differed on other characteristics, referring to the three main variables: ADHD-related traits (high or low), academic achievement level (high, average, or low), giftedness label (yes or no), summing up to a total of 12 vignettes conditions (example vignettes are presented in Appendix B1 and B.2).

Due to the gender-specific morphological structure of the Hebrew language and the higher male-to-female ratio of ADHD diagnosis among elementary school pupils, vignettes were developed using only male-gender form.

### Vignettes’ Validation

Vignettes were distributed to 29 judges, 16 of whom were learning disabilities specialists and or psychologists experienced in the field of ADHD and giftedness. Another 12 were senior teachers or school counselors, and five were neurologists experienced with the diagnosis of ADHD.

Vignettes were divided into two parts. One consisted of a description of either high or low levels of ADHD-related traits exhibited by the child, e.g., “Not once has he not prepared his homework, forgotten to bring his books, or lost his equipment. Often, he has difficulties getting organized at the beginning of class and needs to be reminded of the necessary equipment required for the lesson. During parts of the school day, he has difficulty sitting quietly still.” Based on the description they read, judges were asked to rate on a seven-point Likert scale the likelihood that the child had ADHD.

The second part consisted of characteristics that are either positively or negatively associated with giftedness. For example: “His answers are characterized by a unique thought that advances and contributes to class discussions yet is not always directly related to the matter, showing interest and original thinking with interest in deepening and expanding his knowledge”. Judges were asked to rank on a 7 Likert scale to what degree a specific statement reflects characteristics of gifted children as manifested by school engagement behavior.

Each judge received one of the mixed patterns from both parts of the vignette (i.e., combinations of ADHD [either high, intermittent, or low levels of ADHD-related traits] and giftedness vignettes [compatible or not compatible with characteristics of giftedness]). The vignettes were randomly distributed using Qualtrics software.

The validity of the vignettes was tested using analysis of variance test (ANOVA), where the judges’ rankings served as the dependent variable and ADHD-related traits and characteristics of giftedness served as the independent variables. The results were significant for levels of ADHD-related traits [*F*(2) = 11.877, *p* < 0.001]. Multiple comparisons were conducted using the Tukey measure. A significant difference was found between the judges’ rankings of the likelihood of ADHD in the vignettes describing the high level of ADHD-related traits (*M* = 5.83, *SD* = 1.169) and those describing the low level of ADHD-related traits (*M* = 2.20, *SD* = 1.317). ANOVA results were also significant for giftedness characteristics [F(2) = 5.99, p = 0.022]. A significant difference was found between the judges’ rankings. The rankings for the likelihood that the description in the vignette is compatible with a gifted pupil when characteristics of giftedness were of high compatibility (*M* = 6. 20, *SD* = 0.083), intermediate compatibility characteristic of giftedness (*M* = 4.25, *SD* = 1.89) and a low compatibility characteristic of giftedness (*M* = 3.00, *SD* = 1.00).

### Other Questionnaires

#### Demographic Questionnaire

Participants were asked to state their gender, age, teaching expertise, grade teaching level, school’s education stream association, and socio-economic status of the school rated by the Israeli Ministry of Education ranking code. Responders were also asked if they had previously taken any academic or other courses about ADHD and or giftedness. Participants’ answers are presented in Appendix C.

## Design

The study’s design was a mixed between and repeated measures experimental design. It included three main between-subjects independent variables: ADHD-related traits (low or high) x academic achievement level (low, average, high) x giftedness label (no or yes). These led to the creation of 12 vignette conditions that were predetermined using the vignette validation based on professionals’ ratings. The within-subject measures were teachers’ and counselors’ ratings regarding the likelihood they would refer a hypothetical pupil to ADHD evaluation and the likelihood they would address them during a HISTM.

## Procedure

Each participant was asked to read a short vignette describing a hypothetical pupil in their class. Vignettes only differed regarding the three independent variables. To ensure no bias after reading the first vignette, each participant received only one of 12 randomly allocated by Qualtrics. After reading the vignette, participants were asked to answer questions divided into three sections. First, participants were asked to rate on a 7-point Likert scale (1- defiantly no, 2- unlikely, 3- mostly unlikely, 4- maybe, 5- probably, 6- most probable, 7- definitely): (a) the likelihood they would recommend this pupil to undergo an evaluation for diagnosis of ADHD by an authorized specialist, and (b) the likelihood they would address the pupil during a high-level school team meeting (HISTM). This multidisciplinary professional team meeting was specified to be a high-level attended by the school counselor, school principal, school psychologist, and the school’s inspector from the Ministry of Education. Question order was randomly alternated between participants to control for order confounding effects. Finally, participants were asked to fill out the demographic questionnaire.

All parts of the research were conducted online via the Qualtrics software platform.

**Fig. 1:**
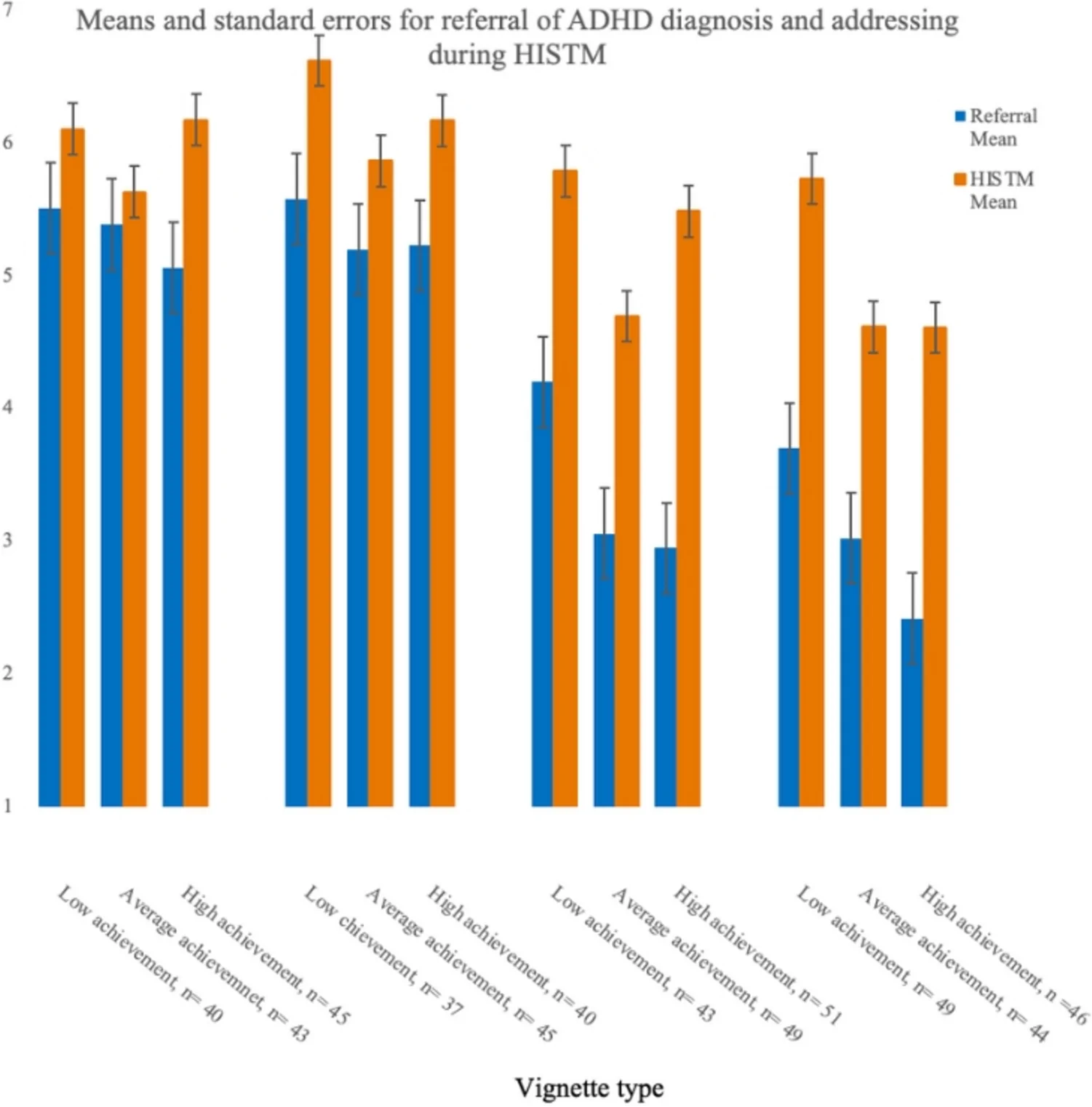
Means and standard errors across conditions

The random allocation to one of the vignettes created 12 experimental conditions. Experimental conditions and the number of participants included in each condition are presented in Fig. 1. Participants could log out at any time. Decide whether to continue their participation later or not to continue answering the questionnaire.

### Statistical Analyses

All statistical analyses were conducted using IBM SPSS software version 27.

#### Planned Tests

Skewness and Kurtosis were assessed to ensure normal distribution of participants’ responses. Descriptive statistics of dependent and independent variables and the correlation between the two dependent variables were computed.

Repeated measures analysis of variance (ANOVA) with significant level set to 0.05 was used to test hypotheses with ADHD-related traits, academic achievement level, and giftedness label as between-subject independent factors and likelihood of referral for evaluation and likelihood for addressing in HISTM as within-subject dependent variables.

To further study interaction effects, one-way univariate tests (ANOVAs) were conducted to determine the likelihood of referral for ADHD evaluation and the likelihood of addressing during HISTM after converting to dummy variables. Planned contrast analyses were conducted to compare among grouping conditions.

#### Missing Data

Missing values from the demographic questionnaire (presented last) are shown in Appendix C. Data from the demographic questionnaire were only used for secondary analyses; therefore, no imputation was employed.

## Results

### Descriptive Statistics

Means, standard deviations, Skewness, and Kurtosis for the likelihood of referral for ADHD evaluation and addressing during HISTM across study conditions are presented in Table 1. Detailed measures of Skewness and Kurtosis for each vignette are presented in Appendix D.

**Table 1:** Descriptive statistics of the dependent variables

				Skewness		Kurtosis	
	N	Mean	SD	Statistic	SE	Statistic	SE
Referral for ADHD diagnosis	532	4.15	1.942	− .066	.106	− 1.226	.211
Referral for HISTM	532	5.56	1.458	− .901	.106	.184	.211

The correlation between the likelihood of referral for ADHD diagnosis and addressing the pupil during a HISTM was large and statistically significant (r = 0.472, p < 0.001).

The 12 research conditions, the number of participants in each condition, and mean and standard deviation across conditions for both referral for ADHD diagnosis and for addressing during a HISM are presented in Fig. 1.

#### ADHD-Related Traits, Academic Achievement Level, Giftedness Label, and Teachers’ Likelihood to Recommend an ADHD Diagnosis and to Address the Pupil During an HISTM.

Three-way repeated measures analysis of variance (ANOVA) was conducted, where the type of decision (referral to an evaluation for a diagnosis of ADHD and addressing in an HISTM) served as a within-subject factor. The between-subject factors were: ADHD-related traits (high/low), academic achievement level (high/average/low), and giftedness label (yes/no). Between-subject ANOVA results are summarized in Table 2.

**Table 2:** Between-subject effects and interactions

	MS	F	df	p	η_p_^2^
ADHD	767.55	290.988	1	< .001	.359
Achievement	103.028	19.53	2	< .001	.070
Giftedness	.153	.058	1	.810	.000
ADHD * Giftedness	2.667	1.015	1	.314	.002
ADHD * Achievement	22.621	4.4288	2	.014	.016
Achievement * Giftedness	3.060	.580	2	.560	.002
ADHD * Achievement * Giftedness	3.257	.617	2	.540	.002

The between-subject analyses yielded significant main effects for ADHD-related traits [F(1, 520) = 290.988, ηp2 = 0.359, p < 0.001] and academic achievement level [F(2,520) = 19.53, ηp2 = 0.07, p < 0.001] but not for the giftedness label[F(1,520) = 0.058, ηp2 < 0.001, p > 0.05].The effect size was considerably greater for ADHD-related traits (η2 = 0.359) than for academic achievement level (η2 = 0.07). Planned contrasts revealed that the likelihood of referral and addressing in a HISTM was higher when academic achievement was low than when it was average (p < 0.001) and high (p < 0.001). For the likelihood of referral for ADHD evaluation, a similar result was observed. Pupils with low academic achievement levels were more likely to be referred than those in the high academic achievement condition (p < 0.001). However, no differences were found between those in the average and high academic achievement level conditions (p = 0.931).

**Fig. 2:**
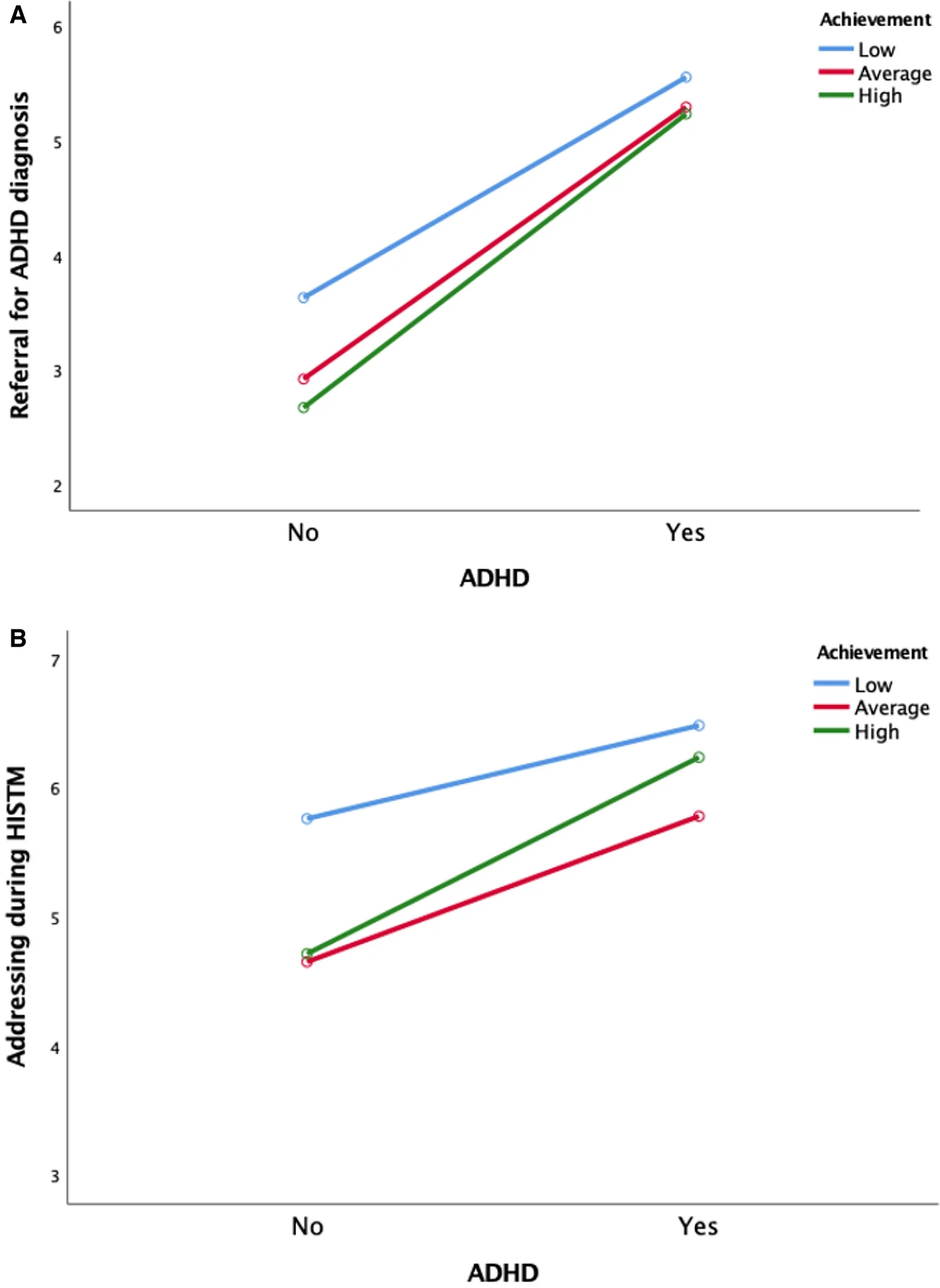
The likelihood of referral for ADHD diagnosis (**A**) and the likelihood of addressing during a HISTM (**B**) as a function of whether ADHD-related traits were indicated and achievement level (N = 523, n for each condition ranged from 77 to 97)

The significant effects of ADHD-related traits and academic achievement level were not stable across each other’s levels, observing an interaction between the two [F(2, 520) = 4.288, η_p_^2^ = 0.016, p = 0.014) (See Fig. 2A and B). Planned contrasts revealed that only when ADHD-related traits were not indicated, low academic achievement level elicited a higher likelihood of referral and addressing during an HISTM (*p* < 0.005). In contrast, when ADHD was indicated, no differences were found across levels of academic achievement (*p* > 0.05).

Results from within-subject ANOVA analysis are presented in Table 3.

**Table 3:** Within-subjects contrasts for main effects and interactions

	MS	F	df	p	η_p_^2^
Type of decision	506.215	350.347	1	< .001	.403
Type of decision *ADHD	89.007	61.601	2	< .001	.106
Type of decision *Achievement	10.369	3.588	2	.028	.014
Type of decision * Giftedness	.929	.643	1	.423	.001
Type of decision *Achievement	.521	.180	2	.835	.001
Type of decision *Achievement * Giftedness	.544	.188	2	.829	.001
Type of decision *ADHD* Giftedness	.702	.486	1	.486	.001
Type of decision *ADHD * Achievement * Giftedness	2.577	.892	2	.410	.003

*Significance values for α = .05

The within-subject analysis for the type of decision revealed a significant effect [F(1, 520) = 350.457, η_p_^2^ = 0.403, p < 0.001] in that likelihood of referral for addressing in an HISTM (M = 5.56, SD = 1.458) was significantly higher than for referral for ADHD evaluation (M = 4.15, SD = 1.942). Interactions were found between type of decision x ADHD condition [F(2, 520) = 61.601, η_p_^2^ = 0.106, p < 0.001] and between type of decision x achievement [F(2, 520) = 3.588, η_p_^2^ = 0.014, p = 0.028]. No other interactions with the type of decision were significant (p > 0.05) (see Table 3).

Planned contrasts revealed that indication of ADHD-related traits significantly increased the likelihood of both referral for ADHD evaluation and for addressing in a HISTM (p < 0.001). Still, the size of the increase was greater for referral for ADHD evaluation than for addressing during an HISTM (1.446 CI [1.254–1.637]) compared to referral for ADHD evaluation 0.825 CI [0.647–1.002]). Another set of planned contrasts revealed that low academic achievement elicited a higher likelihood of referral for diagnosis and addressing in an HISTM (p < 0.005). But the difference between the low and the average academic achievement level was greater for addressing during an HISTM than for referral (0.670, 95% CI [0.454–0.885], and 0.232, 95% CI [0.021–0.442], respectively).

## Discussion

The present study examined the influence of academic achievement level, giftedness label, and ADHD-related traits (i.e., symptoms, school engagement, nonacademic impairments such as: organization, peer relations, injuries, and tardiness) on teachers’ decisions to refer a pupil to evaluation for a diagnosis of ADHD and to address them during a high-level interdisciplinary school team meeting (HISTM).

As hypothesized, results revealed that ADHD-related traits and academic achievement level affected teachers’ likelihood of both the referral of pupils to an evaluation for a diagnosis of ADHD and of addressing them during a HISTM. Teachers were more likely to refer pupils for either an assessment for the diagnosis of ADHD or to address them during a HISTM when ADHD-related traits were indicated and when academic achievement level was low rather than average or high. Effect sizes for ADHD-related traits were larger than for academic achievement level. Hence, ADHD-related traits influenced teachers more than the pupil’s academic achievement level.

The giftedness label did not affect teachers’ referral decision or addressing pupils during an HISTM.

Our findings are congruent with previous research, which found that teachers were able to recognize symptoms related to ADHD among their pupils [9], Perold et al. [38, 54].

These findings have important implications for school settings. Teachers could better serve their pupils if they were aware of the factors that influence them when considering referring a pupil for further professional evaluation. As teachers are more likely to address pupils during an HISTM, offering them an opening for a dialog with other education professionals, they could gain a broader range of insights as to the reasons for the child’s behavior and the right professional to refer the pupil to. By gaining better knowledge of the factors affecting teachers’ decisions, education professionals can be more focused when asking questions to understand the pupils’ and the teachers’ needs and consider alternative reasons for the child’s behavior.

A close-to-ceiling effect was apparent when ADHD-related traits were indicated, yet a floor effect was not evident in the condition where ADHD-related traits were not indicated. In other words, teachers decided to refer some pupils for diagnosis of ADHD even when ADHD-related traits were not indicated. This finding is concurrent with previous research. Several studies suggest that teachers are capable of recognizing ADHD symptoms among their pupils but tend to rate more students as having clinical levels of ADHD symptoms than the actual prevalence in the general population [9], Perold et al. [38, 54]. Since all vignettes in this study described a pupil with social problems, teachers might have attributed these problems to the possibility that the pupil had ADHD. Thus, teachers would rather have a professional clinician assess the child than take the responsibility of not referring the pupil and having the pupil’s disorder not recognized, resulting in the absence of a floor effect when ADHD-related traits were not indicated. Another possible explanation is that these results were affected by a priming effect. Teachers were explicitly asked about a referral for ADHD diagnosis. Therefore, some might have thought that the case presented to them had some elements of ADHD-related traits and thus were primed to indicate they would refer the pupil for diagnosis.

The hypothesis that academic achievement level would influence teachers’ decisions was also supported. Teachers’ rankings were higher when the academic achievement level was low compared to when the academic achievement level was average or high. Previous research found that academic achievement influenced teachers’ referral decisions but not as much as other factors, such as, for instance, pupils’ peer relations [21]. Therefore, some teachers attribute a pupil’s low academic achievement (at least when coupled with social problems) to the possibility that this pupil has ADHD. Either because they know ADHD can affect academic achievement or because ADHD has wrongfully become a popular explanation for why pupils underachieve. An alternative explanation for this result is that children who are not underachieving do not deserve an evaluation, in teachers’ opinion, due to the limited resources.

The hypothesis that an interaction between ADHD-related traits and academic achievement level would affect teachers’ ratings was also confirmed. Figure 2 revealed different patterns for the effects of academic achievement level depending on whether ADHD-related traits were indicated. Planned contrasts demonstrated that when ADHD-related traits were indicated, academic achievement level did not affect teachers’ decisions. Hence, ADHD-related traits were enough to increase the likelihood that teachers would refer a pupil to an evaluation for the diagnosis of ADHD and address them during a HISTM. However, when ADHD-related traits were not indicated, academic achievement level did influence teachers’ decisions. It might be that descriptions of ADHD-related traits caused a near-ceiling effect. Yet, when ADHD-related traits were not indicated and even contraindicated, teachers’ decisions were affected by a pupil’s low academic achievement level. Therefore, when a pupil’s attainment is low, teachers are more likely to consider referring them to a clinician for a formal ADHD diagnosis and addressing them during a HISTM even if the pupil does not consistently and significantly manifest ADHD-related traits.

Results revealed that teachers showed a higher likelihood of addressing the pupil during an HISTM than for referring them to an evaluation for the diagnosis of ADHD. The high and significant gap between participants’ decisions to refer the pupil for diagnosis and to address them during a HISTM may also reflect the hesitance described above or, at the least, a preference towards consulting with their peers. The question of what teachers’ first and foremost course of action would be remains to be further studied by future studies. Future research should consider asking teachers to rank their preferred course of action while controlling for academic achievement levels and ADHD-related traits.

### Effects of Giftedness Label on Teachers’ Decisions

Results revealed that the giftedness label did not significantly affect teachers’ likelihood to refer the pupil to an evaluation for the diagnosis of ADHD or to address them during a HISTM. A possible explanation for these findings is that teachers recognize that giftedness and ADHD may coincide. This conclusion is somewhat concurrent with previous research findings that teachers had reported a greater prevalence of gifted pupils exhibiting clinical levels of ADHD symptoms compared to their non-gifted pupils [46, 55]. If this is the case, it could indicate a positive change in teachers understanding that giftedness does not protect the person from having impairments. However, this result is not in line with other studies that found that teachers attributed less severe ADHD symptoms to gifted-labeled children Rommelse et al. [46] and that gifted children with ADHD tend to be identified later for both ADHD and giftedness. As explained earlier, giftedness may mask ADHD symptoms or make it stick out if a gifted child is underachieving [30]. It may be different when a teacher is given a list of ADHD traits so they can focus on the presence of ADHD better and ignore the label of giftedness.

A different account for these findings, which also has roots in previous research, is that teachers are not aware of the similarities between ADHD-related traits and giftedness-related traits [10, 17, 24, 27, 40]. Therefore, attributing behaviors to ADHD and not to giftedness may lead to a similar referral likelihood. Future studies should consider evaluating this as it might significantly impact gifted students’ well-being.

### Limitation and Implications

This study has several limitations. The main limitation is that teachers were asked explicitly about referral for ADHD. This could prime them to think more thoroughly about the symptoms in a way that would not happen in real life. Still, the results do indicate a level of understanding of the symptoms of ADHD, which may justify a referral for evaluation, whether or not the teacher would have noticed it in real life.

Some limitations relate to the question order, age group, and gender of the pupil described. The two decisions participants were asked to make were presented to them in the same order (first, the likelihood they would refer the pupil to an evaluation for the diagnosis of ADHD and then the probability of addressing the pupil during a HISTM). It may be that question randomization would affect the results. Further, the current study focused on elementary school teachers. Findings might be different among middle school and high school populations.

The vignettes used in this study described a male pupil. Future research should also introduce female or gender-neutral vignettes, as gender is a recognized factor in teachers’ referral decisions (Kypriotaki and Manilitsis [21]).

Another limitation is that teachers’ knowledge and attributions about ADHD and giftedness were not explicitly assessed in this study. Future studies should consider thoroughly examining these factors. By gaining such insight, school psychologists could improve teacher consultation processes, thereby ensuring pupils’ best interests. Finally, the vignettes used in this study described only two options: ADHD-related traits were either indicated or not. Future studies could consider using a more comprehensive range of severity descriptions for ADHD-related traits.

Relevant stakeholders should be aware of the findings discussed above. The main effect of academic achievement level and the pattern revealed within the condition in which ADHD-related traits were not indicated could result in children being referred for ADHD diagnosis even when unnecessary. Although unlikely, and depending on the professional’s expertise, attitude, and ethics, and regarding adhering to formal criteria and guidelines for ADHD diagnosis, such children may be recommended unnecessary treatment for ADHD, including medication.

Teachers and education professionals are primary information providers in the gold standard guidelines for diagnosing ADHD and administrate the implementation of interventions in school settings; they should be aware of the possible causes of the wrong referral or avoiding referral when needed and their implications raised by this study and consider them during their day-to-day work, gaining more knowledge about the traits that justify ADHD evaluation and identifying them in their classroom settings.

## Summary

This study investigated the impact of ADHD-related traits, academic achievement, and the giftedness label on teachers' and counselors' decisions to refer students for ADHD evaluation and to address them during a high-level interdisciplinary school team meeting (HISTM). The results showed that ADHD-related traits had the strongest influence on referral decisions, followed by academic achievement, which increased the likelihood of referral primarily in the absence of ADHD traits. The giftedness label had no significant effect on referral decisions. These findings emphasize the primary role of ADHD-related traits in influencing referral decisions, while academic achievement plays a secondary role. We suggest that improving teachers' understanding of ADHD and other related conditions can enhance the accuracy of referrals and better support students' educational and developmental needs.

## Data Availability

No datasets were generated or analysed during the current study.

## Appendix A

### Summary of Conditions Presented in Vignettes

**Table:**

	ADHD-related traits		Academic achievement level			Giftedness label	
Vignette number	Yes	No	Low	Average	High	Yes	No
1	✓				✓	✓	
2	✓				✓		✓
3		✓			✓	✓	
4		✓			✓		✓
5	✓			✓		✓	
6	✓			✓			✓
7		✓		✓		✓	
8		✓		✓			✓
9	✓		✓			✓	
10	✓		✓				✓
11		✓	✓			✓	
12		✓	✓				✓

## Appendix B.1

### Average Academic Achievement and ADHD-Related Traits are Indicated, and Giftedness Label Exists Vignette

A’ is a gifted pupil with average grades in all subjects attending your class.

He is usually late returning from recess and often busy meddling in social issues he was not directly involved in. During recess, he tends to fall and injure himself. Not once has he come to school with a bandage or a plaster after being wounded during the afternoon.

Not once has he not prepared his homework, forgotten to bring his books, or lost his equipment. Often, he has difficulties getting organized at the beginning of class and needs to be reminded of the necessary equipment required for the lesson. During parts of the school day, A’ has difficulty sitting quietly still. He tends to move around in his chair, changing his sitting positions and sitting on his knees. He fidgets with items on his desk (chews, pencils, and erasers), and in most cases, his workspace is messy. Sometimes, when new material is being taught, he shows interest but tends to lose concentration after a few minutes. Which thereafter draws on some paper or looks outside the window. In some subjects, he is actively involved during the lesson. Sometimes, he seems distracted, finds it difficult to wait his turn to speak, and bursts to answer the question even before it is finished being asked. He tends to debate with the teacher unacceptably and does not let go until he receives the required answer.

From time to time, his answers are characterized by a unique thought that advances and contributes to class discussions yet is not always directly related to the matter. Some of his teachers perceive him as showing interest and original thinking with interest in deepening and expanding his knowledge.

Socially, A’ is not one of the popular kids. During recess, he often walks around on his own. He gets into conflicts with his classmates and some of his teachers, mainly due to his tendency to interrupt what others are saying and their activities. Some teachers have reported they find it difficult to run the class due to his outbursts. On tests and papers, A’s achievements are average, and he does not display any learning difficulties.

## Appendix B.2

### Average Academic Achievement, ADHD-Related Traits not Indicated Giftedness Label Exists Vignette

A’ is a pupil with average grades in all subjects attending your class.

He returns on time from recess; he minds not meddling in social issues he was not directly involved in. During recess, he does not fall and injures himself. Seldom has he come to school with a bandage or a plaster after being wounded during the afternoon.

He prepares his homework and doesn’t forget to bring his books or lose his equipment. He often manages to get organized at the beginning of class and does not need a reminder of the equipment required for the lesson. During the school day, A’, sits quietly still, does not tend to move around a lot in his chair, change his sitting positions, sit on his knees, or fidget with items on his desk (chews, pencils, and erasers). He keeps his workspace tidy. When new material is being taught, he shows interest and continues to do so over time. He manages to concentrate doe does not draw or look outside the window. He is actively involved during the lesson, mostly seems focused, waits his turn to speak, and does not burst to answer the question before it is finished being asked. In seldom cases, he unacceptably debated with the teacher and didn’t let go until he received the required answer.

From time to time, his answers are characterized by a unique thought that advances and contributes to class discussions yet is not always directly related to the matter. Some of his teachers perceive him as showing interest and original thinking with interest in deepening and expanding his knowledge.

Socially, A’ is not one of the popular kids. During recess, he often walks around on his own. He gets into conflicts with his classmates and some of his teachers, mainly due to his tendency to interrupt what others are saying and their activities. Some teachers have reported they find it difficult to run the class due to his outbursts. On tests and papers, A’s achievements are average, and he does not display any learning difficulties.

## Appendix C

### Participant’s Demographic Characteristics

**Table:**

Demographic	N valid data	N missing data		Valid %
Gender				
Female	376			
Male	14			
Age range 18–67 Mean and Standard deviation	M = 41.56, SD = 9.72			
Education level	390	142		
High school	4			1.0
Practical Engineer	1			0.3
BA	199			51.0
Engineer	2			0.5
MA	182			46.7
PhD	2			0.5
School education stream	390	142		
Non religiose State education			265	67.90
Orthodox-state education			105	26.9
Recognized but not official			9	2.3
Independent			5	1.3
Ultra-orthodox			1	0.3
Talmud-Torah			5	1.3
School strength	368	164		
Weak			23	6.3
Medium-Weak			34	9.2
Medium			101	27. 4
Medium-Strong			117	31.8
Strong			93	25.3
Teaching grade	378	154		
First			62	16. 4
Second			40	10.6
Third			73	19.3
Fourth			57	15.1
Fifth			49	13.0
Sixth			53	14.0
School consular			44	11.6
ADHD academic courses				
Yes			310	80.3
No	386	146	76	19.7
Gifted studies	389	143		
Yes			32	8.2
No			357	91.8
Nonacademic ADHD courses	387	145		
Yes			227	58.7
No			160	41.3
Nonacademic gifted courses	388	144		
Yes			96	24.7
No			292	75.3

## Appendix D

### Skewness and Kurtosis Across Vignette Type

**Table:**

Vignette type	n	Referral for diagnosis				Team Meeting			
		Skewness		Kurtosis		Skewness		Kurtosis	
		Statistic	SD error	Statistic	SD error	Statistic	SD error	Statistic	SD error
ADHD + , gifted label, low Achievement	40	− 1.104	0.374	0.607	0.733	− 1.369	0.374	0.525	0.733
ADHD + , gifted label, average achievement	43	− 0.735	0.361	0.280	0.709	− 0.755	0.361	− 0.585	0.709
ADHD + , gifted label, high achievement	45	− 0.735	0.354	0.295	0.695	− 0.934	0.354	0.108	0.695
ADHD + , no label, low achievement	37	− 1.153	0.388	1.037	0.759	− 2.359	0.388	5.221	0.759
ADHD + , no label, average achievement	45	− 0.494	0.354	− 0.393	0.695	− 0.883	0.354	0.091	0.695
ADHD + , no label, high achievement	40	− 0.458	0.374	− 0.903	0.733	− 1.292	0.374	0.987	0.733
ADHD** − **, gifted label, low achievement	43	0.167	0.361	− 1.057	0.709	− 0.764	0.361	− 0.102	0.709
ADHD** − **, gifted label, average achievement	49	0.357	0.340	− 0.465	0.668	− 0.355	0.340	0.334	0.668
ADHD** − **, gifted label, high achievement	51	0.682	− 0.333	− 0.794	0.656	− 0.473	0.333	− 0.714	0.656
ADHD** − **, no label, low achievement	49	0.470	0.340	− 0.571	0.668	− 0.748	0.340	− 0.688	0.688
ADHD** − **, no label, average achievement	44	0.766	0.357	− 0.220	0.702	− 0.328	0.357	− 0.154	0.702
ADHD** − **, no label, high achievement	46	1.530	0.350	2.090	0.688	− 0.495	0.350	− 0.361	0.688
